# Care Beyond the Bedside: Creating Space for Families of Hospitalized Children with Medical Complexity

**DOI:** 10.3390/children12070917

**Published:** 2025-07-11

**Authors:** Claire E. Wallace, Patrick G. Hogan, Nicholas A. Holekamp

**Affiliations:** Ranken Jordan Pediatric Bridge Hospital, 11365 Dorsett Road, Maryland Heights, MO 63043, USA; patrick.hogan@rankenjordan.org (P.G.H.); nick.holekamp@rankenjordan.org (N.A.H.)

**Keywords:** children with medical complexity, neurodevelopment, pediatric post-acute care, prolonged hospitalization, caregiver engagement

## Abstract

Prolonged hospital stays separate children from their families and adversely impact the well-being of both. Children with medical complexity (CMC) often have long hospital stays and sometimes spend months to years missing their childhoods, often alone in their rooms. Caregivers of CMC must navigate many barriers to discharge during long hospital stays, which further strains the family system. In this review, we summarize the developmental vulnerabilities of chronically hospitalized CMC and propose that the hospital environment itself confers additional risk for poor neurodevelopmental outcomes. We will discuss the opportunities for pediatric post-acute care (PPAC) hospitals to create spaces where medical treatment, developmental recovery, and family integration in care can exist simultaneously. We then describe how the Care Beyond the Bedside model developed by one PPAC hospital aims to diminish the detrimental effects of prolonged hospitalization on CMC and their families by prioritizing developmental opportunity alongside medical stability. Critical components of this care model are patient and family spaces designed for community, safety training to supervise patients away from the bedside, and investment in staffing and programming to support the model. This care model acknowledges that play and healing are inextricably linked and that children develop best when they are out of bed, participating in life with their families.

## 1. Introduction

In 1947, endocrinologist and hematologist Richard Asher strongly asserted to the medical community in “The Dangers of Going to Bed” [1] that hospital beds and graves should be thought of in the same way, enumerating many of the bodily systems which can be harmed by prolonged stays in bed. In the decades since, research has supported Asher’s assertion that prolonged bed rest can hinder recovery due to limited activity and mobility [2,3]. For children with medical complexity (CMC), prolonged hospitalization can become an unfortunate reality. The impact of prolonged hospitalization and time confined to bed can be developmentally disastrous, considering the months to years CMC can spend deprived of developmentally rich environments [4]. Few studies have attempted to improve, or even quantify, the time hospitalized children spend in bed [5].

In this article, we describe the problem of prolonged hospitalization and propose a transformation of the inpatient hospital experience, removing or reducing some of its inherent insults to development. We then describe the unique care model at Ranken Jordan Pediatric Bridge Hospital, a post-acute care hospital serving CMC and their families. The model, Care Beyond the Bedside, prioritizes neurodevelopment, family engagement, and play as soon as possible during a child’s hospitalization, with a goal of reducing the impact of hospitalization on child development and family well-being.

## 2. Illustrative Case—Part I

Forest was 14 months old when he was diagnosed with a spinal cord tumor with cervical and thoracic extension that required emergent surgical resection due to rapidly progressing respiratory failure. After six weeks of acute care support, he achieved relative medical stability with a gastrostomy, tracheostomy, and full-time mechanical ventilation for his quadriplegia. However, it would take another nine months to get Forest, his family, and his residence ready for safe discharge home.

## 3. The Problem of Prolonged Hospitalization

Children are increasingly surviving stays in neonatal and pediatric intensive care, creating a significant need for services that address the physical, cognitive, social, and emotional needs of these survivors and their families [6]. Even short-term exposures to the intensive care environment can carry serious developmental consequences beyond the initial injury. Children who develop pediatric post-intensive care syndrome (PICS-p) [7] can suffer developmental consequences across domains that extend far past their ICU stays [8,9]. Children who require prolonged hospitalization are particularly at risk for further developmental delay [10,11], an experience common among technology-dependent CMC. Prolonged hospitalizations, often exacerbated by significant discharge barriers [12], expose children to lengthy periods in suboptimal developmental environments, further aggravating the health and developmental disparities of CMC. Insults to development inherent in hospitalization include restricted independence, chronic and acute stressors, disrupted parent–child attachment, limited interaction with peers, and disrupted sleep [12].

Mobility is one developmental domain significantly impacted by prolonged hospitalization. The “ON Time Mobility” framework, which centers mobility as a human right, argues that mobility enables children to discover, build relationships, and shape their lives [13], consistent with United Nations designations mandating the Rights of Children and Persons with Disabilities [14,15]. Some hospitals have designed interventions that reflect the importance of mobility, focusing on measuring or increasing mobilization events for children hospitalized even for short durations in the pediatric intensive care unit (PICU) [16,17,18,19]. For example, the PICU Up! intervention focuses on increasing both in-bed therapies and mobility activities (sitting at edge of bed, sit to stand, transfer, ambulation, and play) in the first three days after PICU admission [16]. A systematic review suggests that this type of early mobilization in the PICU is both safe and feasible, with potential benefits in both the short-term and the long-term [20]. For CMC requiring prolonged hospitalizations, the benefits of facilitating mobility may be even more impactful given the cognitive developmental gains that accompany independent mobility [21].

Social relationships are another domain significantly disrupted by prolonged hospitalization. Isolation from primary caregivers, other family members, and peers is often part of the hospital experience for chronically hospitalized CMC. Clear pathways between social connection and health, established among adults [22,23], likely also impact the vulnerable and developing pediatric brain. Importantly, meta-analyses suggest that true social integration has more health benefits than one-sided received support [24]. For hospitalized children, naturally occurring social support may be more successful than support provided by hired personnel performing caretaking, nursing, and medical activities. To incorporate social connection in the hospital, we can leverage evidence-based interventions that facilitate psychosocial support for patients, including involving family or other caregivers in treatment and initiating group therapies [25].

Acknowledging the problem of prolonged hospitalization is critical to addressing it. Alongside ongoing neurodevelopmental research on the impact of prolonged pediatric hospitalization [4,10,11,26], ethics research is evaluating this topic [27,28]. One study assessed the dilemmas reported by neonatal nurses and physicians caring for newborns with extremely long hospitalizations (>6 months). A primary theme identified was their concern for children’s ability to “create a normal life” [28]. Respondents worried about children not learning basic developmental skills, like how to sit, crawl, or talk, and described babies left for lengthy periods of time, alone in their cribs. While pediatric providers are unlikely to argue that prolonged hospitalization is good for child development, deepening our understanding of the deleterious effects of long-term hospitalization on child and family health is a high priority research target. As PICU and neonatal intensive care unit (NICU) survivorship increases, those responsible for the prolonged hospitalization of children are obligated to consider the long-term consequences of lengthy exposures to the hospital environment.

## 4. Supporting Caregiver Involvement

Hospitalization separates children from familiarity and from family [29]. The unnatural separation of CMC from their caregivers during prolonged hospitalizations can strain the caregiver–child relationship on both sides and can impact child development and family well-being. In the NICU setting, parental visitation and holding have been linked with positive early neurobehavioral outcomes for infants, including quality of movement, stress level, arousal, and excitability [30]. However, caregivers of children hospitalized for prolonged periods tend to become less engaged over time [28,30,31,32,33]. In a study of infants hospitalized in the NICU for >6 months, staff report that 23% of parents were either never involved or did not stay involved throughout the hospitalization [28]. Other NICU studies have reported that the number of days parents are present and hours they visit decrease as length of hospitalization increases [30,33].

To fully understand factors that contribute to caregiver visitation and engagement in care, we must look to barriers faced by caregivers of CMC. In the pediatric post-acute care (PPAC) hospital setting, caregivers of CMC are less likely to visit after multiple admissions, when they must travel long distances, if supported by public insurance, and if Child Protective Services has been involved [31]. Caregiver engagement research is working to determine activities that encourage active caregiver involvement while the child is hospitalized [28], identify factors that promote or prevent engagement and visitation, and measure stress and coping skills of caregivers [31].

Caregiver interviews can elucidate areas for improvement in the hospital experience. Parents of children hospitalized between 1–15 months, interviewed as part of a hospital program focused on young patient development, want positive and ordinary experiences for their child while hospitalized, appreciate emphasis on child development during otherwise complex medical care, and derive confidence from appropriate developmentally focused education, which allows them to envision caring for their child at home [34]. Parents of children discharged home with invasive mechanical ventilation echoed the sentiment that family-centered approaches to caregiver training could decrease parental stress related to learning complex medical care [35]. By supporting families experiencing the barriers described above, hospitals can generate a positive feedback loop—when caregivers are more present, neurodevelopmental outcomes improve; in turn, when neurodevelopment is a key target during hospitalization, it helps caregivers remain connected and engaged with their medically complex children.

## 5. The Role of Pediatric Post-Acute Care Facilities

Barriers to discharge among CMC are well documented [12,36,37] and carry significant costs. Across 44 children’s hospitals, 15% of CMC hospitalizations are long length of stay (>10 days); these long stays account for 61% of hospital days and 62% of hospital costs for CMC [38]. Within 43 NICUs, 24% of NICU days occur after the point at which NICU patients do not require or receive NICU-level care, demonstrating non-medical delays of discharge [39]. Beyond resource costs, hospitalizing children for longer or in more restrictive environments than are medically necessary carries developmental costs, particularly for CMC and survivors of critical illness [4,40,41]. When their prolonged hospitalizations occur in developmentally suboptimal spaces, both before and after medical readiness for discharge, it exacerbates developmental disparities and increases stress on families. If home is not an option, the focus should be on optimizing the location in which less acutely ill children are treated [42].

PPAC facilities have evolved to bridge the gap between the acute care hospital and home [6,43,44]. The patient population served by PPAC facilities is institution-dependent, but patients typically are medically complex with tracheostomy/ventilator dependence and/or parenteral nutrition needs. PPAC facilities are uniquely positioned to meet children’s and families’ needs in a different way than acute care hospitals. Because patients have typically achieved a degree of medical stability prior to transition to a PPAC facility, the focus can shift from survival to recovery and from developmental stagnation to developmental progress. PPAC facilities serve children who continue to have medical goals that prevent discharge and often have other barriers to transitioning home [44]. Transitioning to post-acute care can provide an earlier intervention option to combat the neurodevelopmental insults that accrue over time in developmentally suboptimal environments.

## 6. Promoting a Culture That Values Developmental Opportunities

As collective understanding of the impact of prolonged hospitalizations on CMC increases, disseminating that knowledge to the medical community will be critical in shifting the culture of care. Interventions that aim to increase mobilization, for example, cite culture, collaboration, and institutional support as vital to success [16,20,45]. In the PICU Up! study, new barriers to mobilization were identified after implementation, including lack of appropriate seating devices and positioning materials and inadequate staffing [16]. This suggests a lack of awareness and institutional readiness that required intentional intervention to uncover. In adult care settings, interventions to reduce sedentary hospital behaviors have required innovation not just in equipment but in mindset and the hospital environment itself [45]. Preventing isolation and inactivity inherent to the hospital environment requires an active, culture-driven philosophy. PPAC facilities are well positioned to implement out-of-bed interventions and turn the focus of hospitalization to child development, but these interventions require a significant cultural shift for the entire institution.

## 7. A Unique Care Model—Ranken Jordan Pediatric Bridge Hospital

Ranken Jordan Pediatric Bridge Hospital is a 60-bed PPAC hospital for CMC in Missouri consisting of two inpatient units (https://rankenjordan.org/ accessed on 8 May 2025). On the 0–5-year-old patient unit, 64% of patients have gastrostomy tubes and 34% have tracheostomies; average total stay is 167 days, often in addition to months in a NICU or PICU before transitioning to Ranken Jordan. On the 6+ year-old patient unit, 31% of patients have gastrostomy tubes, 14% have tracheostomies, and they spend an average of 61 days at Ranken Jordan. While some PPAC facilities only care for children once medically stable and ready for discharge, Ranken Jordan serves patients with ongoing medical and therapy goals and continues to provide care until a safe discharge plan is in place.

Our unique care model—Care Beyond the Bedside—is based on the philosophy that play and healing are inextricably linked and that children develop best when they are out of bed, participating in life with their families. Care Beyond the Bedside aims to address a blind spot in pediatrics—that the hospital environment itself contributes to the developmental delays common among chronically hospitalized children and that the hospital environment can be changed to optimize rather than suppress child development.

### 7.1. Physical Space

If children who are hospitalized for months to years at a time are to make developmental gains, they must be out of their beds. As summarized above, mobility has important implications for both general development and acute post-injury/illness recovery, even in the first few days of an ICU stay. To provide Care Beyond the Bedside in its richest form, Ranken Jordan designed every space with mobility, play, and social interaction in mind. Like most children’s hospitals, color and natural light are key design elements. Unlike most children’s hospitals, the rooms themselves have no televisions and house more than one patient. There are on-unit play spaces to draw children out of their beds, out of their rooms, and into community with their peers and family members. Open play spaces, an indoor pool, indoor and outdoor playgrounds, a rock-climbing wall, and multipurpose event spaces are designed to engage children and families in special activities throughout the week. Two classrooms engage children in group programming with early education and socialization goals. The horticulture program affords patients the opportunity to maintain a garden on the outside grounds, while an in-house music therapist and resident artist provide daily enrichment away from the bedside. Open event spaces draw in magicians, petting zoos, scientists, and dancers. To truly reduce the impact of hospitalization on development, hospitals must be intentionally designed around child development and family engagement.

### 7.2. Safety

In acute care settings, avoiding unnecessary risk of harm is a primary justification for keeping CMC, especially those with artificial airways, in their rooms. Research supports a strong safety profile for mobilization and rehabilitation, even in the PICU [46]. The same must hold for CMC in the post-acute care setting. Therefore, safety and emergency preparedness, including a response capability that mimics what is available at bedside, are critical components of Ranken Jordan’s care model.

The emergency response team (ERT), which trains with “mock codes” regularly, responds to any medical emergency, anywhere on the hospital grounds. All patient care employees wear Vocera Smartbadges, and pull cords installed in every patient space allow both employees and families to quickly call for the ERT. With this safety system in place, patients with artificial airways can leave the clinical units without a respiratory therapist or nurse present. Before families and visitors can take patients off the clinical units, they participate in Ticket to Ride, a program in which bedside staff teach safe patient transport and assess competence of caregivers’ response to emergencies.

Ventilator carts, airway backpacks with emergency supplies, Bluetooth pulse oximeters linked wirelessly to a centralized monitoring capability (SafetyNet), portable oxygen, and portable suction machines are issued to every patient with an artificial airway. Everything that would be available at the bedside accompanies the child wherever they go, and composite screens throughout the building ensure that medical personnel have continuous awareness of each child’s heart rate and oxygen saturation wherever the child is on the campus. Investing in these technologies that allow for mobility away from the bedside is a critical component of Care Beyond the Bedside.

The infection prevention team maintains patient safety while accommodating enhanced opportunities for child development. Single-patient rooms and isolation are common approaches in acute care settings. However, as physical isolation is antithetical to child development, the focus in this model is on isolating the contagion rather than the child. Daily symptom screening identifies patients who need further monitoring or in-house testing before participating in group activities, and staff prioritize hand hygiene for the patients themselves, especially during group activities. When children diagnosed with a viral illness are in the contagious window but appear well, specific precautions allow them to utilize dedicated spaces inside and outside the hospital rather than remaining in their beds, in their rooms. Ill patients are often cohorted together, both in their physical rooms and in select group activities. This approach reduces the time an infection compels a child to spend alone, without significantly increasing exposure risk.

### 7.3. Staffing and Programming

We have learned that more trained and engaged staff equals more children out of their beds. Like many children’s hospitals and rehabilitation facilities, Ranken Jordan has teams of child life specialists, recreational therapists, and physical/occupational/speech therapists who regularly engage children out of their beds. A newer role, the patient play associate, fills a need for trained staff members whose sole focus is play across the developmental spectrum. A uniting force among all employees, including respiratory therapists, nurses, and doctors at the bedside, is the culture of Care Beyond the Bedside. Play is a fundamental right of all children [14] and a core value across the institution, included in employee performance reviews and even written on the walls. It is not considered a luxury or an add-on for patients’ care—it is an expectation.

A strong institutional value of play must be paired with extra staff training to ensure all staff can keep patients medically safe away from the bedside. New direct patient care employees complete a minimum of 4 h of hands-on training in respiratory safety and safe patient handling. All patient care employees also complete mandatory annual education via online modules and in-person training, which address the logistical challenges of Care Beyond the Bedside, including feeding tube management, transferring patients, prevention of medical device pulling, and reading monitor output from ventilators/pulse oximeters/feeding pumps.

A yearly time–motion study assesses key elements of Care Beyond the Bedside for young patients ages 0–5 [5]. By following children during their waking hours (7 a.m.–7 p.m.), the quality improvement initiative measures patients’ exposure to developmentally rich environments, including where they are in the hospital, who they are with, and what they are doing. In the time–motion study’s first year, patients spent 44% of their awake hours out of bed. Time out of bed was not significantly different between ventilator-dependent and non-ventilator-dependent patients.

A multidisciplinary committee at Ranken Jordan spearheaded several initiatives to further increase time out of bed, including the optimization zone (OZ), on-unit play spaces, and patient play associates. The OZ program brings young children into specialized group programming to support their development alongside peers. Since the inception of OZ in 2022, more than 200 patients have participated in the program, totaling over 9000 h of additional engagement in developmentally rich spaces. OZ extends Care Beyond the Bedside by providing another dedicated opportunity for children to integrate with their peers as well as with their families in a space designed to be play-forward.

### 7.4. Enhancing Family Health and Well-Being

From the special events to the open play spaces to the safety systems across the hospital, Care Beyond the Bedside aims to facilitate family togetherness. While the home environment will always be the most developmentally enriching space for children to grow up, chronically hospitalized children and their families deserve hospital spaces that optimize their development. By caring for their medically complex children in spaces like the outdoor playground and the dining room, families can engage with their children in routine daily activities to build their comfort level with this complex care before they transition home. Likewise, community integration trips bring patients and their families away from the hospital to practice navigating community spaces together. As prolonged hospitalization reduces caregiver engagement over time, one study proposed creating activities that encourage parents’ active involvement in the child’s care, both in and out of the hospital [28]. By helping family members see themselves more like parents of children and less like caregivers to patients, Care Beyond the Bedside responds to that call.

Another crucial component of family support is addressing mental health and other resource needs [47]. Each family has a social worker, care coordinator, and pediatric psychologist automatically involved as members of the care team. Child life specialists and a chaplain support families through services such as diagnosis education for siblings, memory making, and grief/bereavement support. Including these members of the care team from the beginning of each admission normalizes the universal need for mental health and resource navigation among families of CMC.

Care Beyond the Bedside also seeks to reduce some of the burden on caregivers by improving children’s functioning across developmental domains. Children who have experienced more stimulating spaces and group programming have improved tolerance for more stimulating environments after discharge. Children who have attended OZ have learned the structure and expectations of a preschool setting before they attend their first days of preschool or daycare at home. Children who have had more opportunities to freely move their bodies and explore their surroundings can develop more muscle strength and coordination, often improving their respiratory capacity. One emerging research question is whether developmental optimization during hospitalization can help CMC become less complex.

### 7.5. Implementation

Care Beyond the Bedside is fundamental to our mission, and its implementation is enabled via foundational relationships with the state Medicaid agency and state leaders. Establishing Ranken Jordan as a pediatric specialty hospital, an organizational licensure that matches the inpatient services we provide, has been crucial to our financial viability. We continually work with our state to evaluate how Ranken Jordan can be the best steward of state funds to meet the needs of CMC. Transitioning patients from tertiary care ICU environments into a post-acute care hospital environment is a cost saving measure [48]. Ensuring families of CMC are adequately prepared for their transition home can also diminish the cost of their high healthcare utilization, demonstrated by decreased emergency room visits and re-admissions [49]. Donor dollars raised through our Foundation strengthen and support components of Care Beyond the Bedside not funded by traditional mechanisms of the healthcare reimbursement system, such as the optimization zone and the patient play associate role.

Having a strong financial support structure enables Ranken Jordan to implement Care Beyond the Bedside in a robust fashion, with adequate staffing, physical space, and safety systems. There is no one stand-alone element of Care Beyond the Bedside that can be pulled into other settings providing care for CMC, as the model truly relies on a combination of physical space, staffing, and culture. However, institutions seeking to make changes can start with raising institutional awareness of the significant risk of developmental delays that CMC face during prolonged hospitalization and reconceptualizing the hospital environment itself as a modifiable factor in these children’s care.

## 8. Illustrative Case—Part II

Forest was admitted to Ranken Jordan Pediatric Bridge Hospital 6 weeks after his initial tumor resection and spent the next 9 months navigating autonomic dysreflexia, dysphagia, temperature dysregulation, neurogenic bowel, and obstructive bronchiectasis while also fully immersed in the Care Beyond the Bedside model. He learned to communicate with an eye gaze device, re-learned to eat and drink by mouth, built muscles for head control and sitting balance, formed relationships with peers, learned preschool routines, and eventually found his voice using a speaking valve. Forest’s family spent those nine months recalibrating their lives as parents of a child with medical complexity, becoming experts in his medical care, advocating for his overall support needs, and re-working family routines to accommodate Forest’s needs.

**Figure 1:**
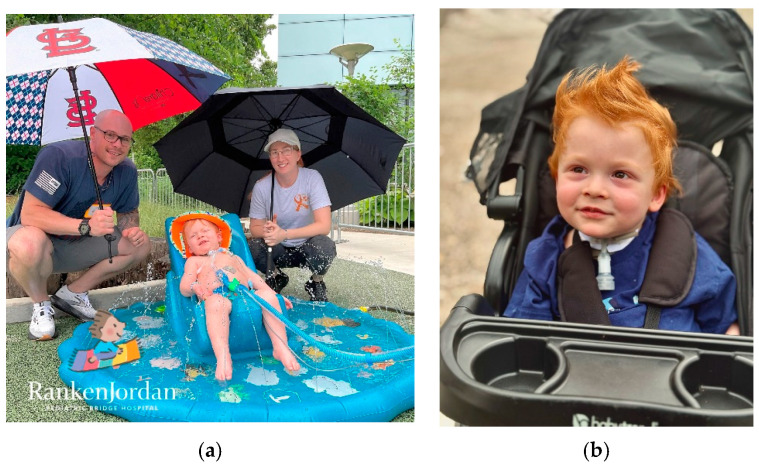
Forest and his family, (**a**) Forest and his parents living Care Beyond the Bedside during "water day" in OZ at Ranken Jordan (**b**) Forest at home, riding in his stroller.

Care Beyond the Bedside at Ranken Jordan created spaces where Forest and his parents could re-establish themselves as a family. Forest spent most of his waking hours out of his bed and out of his room, optimizing his development in ways that would not have been possible in the acute care setting (Figure 1a). As Forest approached medical discharge readiness, it became clear that it could take months or longer to establish adequate private duty nursing at home. Forest’s family felt prepared to care for him at home without additional support due to daily practice with administration of medications and meals, suctioning and other aspects of his ventilatory support regimen, and transfers in and out of his bed and wheelchair. The extensive, reality-based training in combination with maximizing time together with their child gave them the skill and confidence to care for his significant medical complexity on their own at home where he could continue his journey, thriving rather than merely surviving (Figure 1b).

## 9. Future Directions

As the complex care community researches the impact of prolonged hospitalization on child development, complementary research should begin to identify solutions. Ranken Jordan will continue to optimize the Care Beyond the Bedside model to maximize children’s time out of bed and to measure the impact of the care model on children’s development. Acute care hospitals can increase developmental opportunities during a child’s initial hospitalization and expedite the timing of transition to PPAC facilities that, in turn, should prioritize getting children out of bed and into developmentally rich environments.

Given the importance of genuine family engagement in a child’s recovery, hospitals should prioritize increasing time together with caregivers while the child is receiving the level of medical care that cannot yet be managed at home. In some settings, caregivers are limited to brief overnight stays to demonstrate knowledge of their child’s care just prior to discharge. In other settings, sleeping quarters are available nearby through organizations such as the Ronald McDonald House. Newer developments have begun to include suites that combine a hospital room with a room for a family member to stay, further increasing caregivers’ comfort and proximity to the child. Combining the hospital room and the home, complete with bedroom(s), living spaces, and amenities, would be the logical extension of this idea and would afford the whole family maximal time physically with the patient, either for brief stints in preparation for discharge or for the entirety of the stay. This extreme iteration is currently anathema to acute care medicine. True implementation would require innovative problem-solving to overcome the many logistical and economic hurdles it presents. Despite the challenges, we as complex care providers are compelled to continue exploring innovative models of care to bring children and their families together. Family is central to childhood. While discharge home remains the ultimate goal, childhood cannot wait for discharge.
